# Case of Alopecia

**Published:** 1849-11

**Authors:** 


### *Case of Alopecia.*

**To the Editor of the Boston Medical and Surgical Journal.**

Dear Sir,—Please publish, as a professional curiosity, the following extraordinary case of alopecia.

The subject, a child of Mr. ———, a lovely, dark-haired little girl, aged 11, in perfect general health, although suffering at times from palpitation of heart—cause, it is feared, organic—in September last perceived patches of baldness encroaching upon lateral, posterior and vertical regions of scalp, for which the mother, after various domestic applications, had the head perfectly shaved—probably a very appropriate remedy. Some two months elapsed, and *no hair re-appearing,* my professional attention was called to the case. Found the scalp, (with the exception of some dozens of scattered hairs one-eighth of an inch long, of original color and partially loosened from their attachment), in a perfectly nude state, exhibiting a whitened, polished surface,—not the least vestige of natural appearance remaining. Prescribed, generally—exercise, open air, &c.; and locally, bathing with cold water, brisk frictions daily with dry table salt, to which was superadded, after a few weeks, a solution of tartrate of antimony, ʒj. to aqua ℥iv., until slight irritation supervened.

In about six weeks, a soft silvery down began to clothe the whole surface previously occupied by the natural hair. The mother, painfully anxious for the result, inclined to reproach herself for having the head shaved without previous consultation; her anxiety, however, was easily removed by the fact now exhibited to her, of the commencing depilation of both eyebrows and lids. Thus appearances remained for months, the *down* rather increasing in thickness and coarseness, and some hairs upon the vertex becoming firm and inclined to grow, to the length of several lines.

The case has been under professional supervision from incipiency, and yet presents no more natural aspect; in fact, now there is almost a total absence of downy covering to the scalp, the eyelashes and eyebrows, however, having been replaced with silvery hairs of nearly their ordinary length and firmness.

In addition to the above phenomena, we have now a corresponding change in the color of the iris; its hue, formerly dark hazel, gradually assuming the pale pinkish aspect of the natural Albiness. Extraordinary! is it not? and so far as my observation goes, unique—although I have witnessed patches of dark hair changed to grey—and without any apparent assignable cause; both mother and father, and all the other children, having dark, thick, glossy hair!

*Quere*—what, if any thing, is to be done? As an antiquated patient of mine, one of those everlastingly complaining individuals not unfrequently met with in professional intercourse, after expending her utmost physical force in the narration of a host of symptoms, covering the entire person from head to heel, used to say, "Now, Doctor, you're puzzled, ain't ye?" If not, say so, and oblige your old correspondent and friend,

PROVIDENCE, R. I., July 15, 1849. J. MAURAN.

